# Identification of a prognostic evaluator from glutamine metabolic heterogeneity studies within and between tissues in hepatocellular carcinoma

**DOI:** 10.3389/fphar.2023.1241677

**Published:** 2023-10-26

**Authors:** Jie Bao, Yan Yu

**Affiliations:** ^1^ Digestive System Department, The First Affiliated Hospital of Zhengzhou University, Zhengzhou, China; ^2^ Department of Infectious Diseases, The First Affiliated Hospital of Zhengzhou University, Zhengzhou, China

**Keywords:** hepatocellular carcinoma, single-cell sequencing analysis, glutamine metabolism, machine learning algorithm, risk model, prognosis, immunotherapy

## Abstract

**Background:** The liver is the major metabolic organ of the human body, and abnormal metabolism is the main factor influencing hepatocellular carcinoma (HCC). This study was designed to determine the effect of glutamine metabolism on HCC heterogeneity and to develop a prognostic evaluator based on the heterogeneity study of glutamine metabolism within HCC tumors and between tissues.

**Methods:** Single-cell transcriptome data were extracted from the GSE149614 dataset and processed using the Seurat package in R for quality control of these data. HCC subtypes in the Cancer Genome Atlas and the GSE14520 dataset were identified via consensus clustering based on glutamine family amino acid metabolism (GFAAM) process genes. The machine learning algorithms gradient boosting machine, support vector machine, random forest, eXtreme gradient boosting, decision trees, and least absolute shrinkage and selection operator were utilized to develop the prognosis model of differentially expressed genes among the molecular gene subtypes.

**Results:** The samples in the GSE149614 dataset included 10 cell types, and there was no significant difference in the GFAAM pathway. HCC was classified into three molecular subtypes according to GFAAM process genes, showing molecular heterogeneity in prognosis, clinicopathological features, and immune cell infiltration. C1 showed the worst survival rate and the highest immune score and immune cell infiltration. A six-gene model for prognostic and immunotherapy responses was constructed among subtypes, and the calculated high-risk score was significantly correlated with poor prognosis, high immune abundance, and a low response rate of immunotherapy in HCC.

**Conclusion:** Our discovery of GFAAM-associated marker genes may help to further decipher the role in HCC occurrence and progression. In particular, this six-gene prognostic model may serve as a predictor of treatment and prognosis in HCC patients.

## Introduction

Liver cancer is a malignant tumor with the fastest increasing mortality and has become the second leading cause of cancer-induced death (Siegel et al., 2023). The main types of liver cancer include hepatocellular carcinoma (HCC) and cholangiocarcinoma. HCC is the most common form of liver cancer, accounting for 90% of the cases (Llovet et al., 2021). Most HCC cases occur in Asia, and the main known risk factors relevant to HCC are viruses (chronic hepatitis B and C), metabolism disorders (diabetes and non-alcoholic fatty liver disease, or NAFLD), toxicity (alcohol and aflatoxin), and immune system-related diseases (Chakraborty and Sarkar, 2022). The United States Food and Drug Administration approved liver cancer treatment options involving multi-kinase inhibitors (lenvatinib, regorafenib, ramucirumab, and cabozantinib), immune checkpoint blockades (ICBs) (pembrolizumab and nivolumab), and combination therapies, such as atezolizumab along with bevacizumab (Butt and Baytas, 2023). Although substantial breakthroughs have been made in systemic treatment, the mortality rate of HCC has remained high owing to drug resistance and frequent relapse (Chidambaranathan-Reghupaty et al., 2021; Chen et al., 2023). Individual and intratumoral heterogeneity greatly affects the recurrence and drug resistance in patients with HCC (Zhang et al., 2020). Therefore, understanding the causes, characteristics, and consequences of HCC tumor heterogeneity is necessary to guide clinical practice and improve survival.

The liver is the main metabolic organ in the human body (Chakraborty and Sarkar, 2022). Metabolomics and metabolite profiling of HCC have been in the spotlight for cancer diagnosis, monitoring, and therapy (Ganesan et al., 2022). Small molecular metabolites play an important role in biological systems and are attractive candidates for understanding the HCC phenotype (Wang et al., 2013). The catabolism, anabolism, and transport of glutamine are essential for the survival and development of HCC (Altman et al., 2016). Targeting glutamine metabolism is a promising anti-cancer therapy. Several inhibitors targeting glutamine metabolism have been created, such as allosteric inhibitors of kidney-type glutaminase (GLS) and CB-839 (telaglenastat), which have entered different stages of clinical trials for cancer treatment (Yang et al., 2021). However, it is noteworthy that the metabolism of glutamine in cancer is highly heterogeneous. Even in tumors in specific organs, different cancer subtypes have different patterns of glutamine metabolism (Cluntun et al., 2017). Therefore, exploring the glutamine metabolism model will help in the accurate classification and patient stratification of HCC.

In this study, we identified, for the first time, cellular subtypes of HCC based on glutamine metabolism-related genes and explored the heterogeneity of these genes within HCC tumors and among tissues. In addition, we classified the HCC subtypes according to the genes in the glutamine metabolic pathway and used six different machine learning methods to construct a risk evaluator that explored the relationship between potential factors affecting HCC prognosis (clinicopathological features, somatic mutations, tumor microenvironment (TME) indicators, signal pathways, and indicators of immunotherapy response) and different glutamine metabolic subtypes of HCC.

## Materials and methods

### Single-cell RNA sequencing dataset and the RNA-seq dataset of HCC samples

Sample data from the GSE149614 dataset were extracted from the GEO database. The dataset included 71,915 single-cell transcriptome data from four HCC-related tissue types: non-tumor liver (NTL, *n* = 8), primary tumor (PT, *n* = 10), portal vein tumor thrombus (PVTT, *n* = 2), and metastatic lymph node (MLN, *n* = 1). There were three source databases of RNA-seq data for HCC samples: the Cancer Genome Atlas (TCGA) (title = "https://portal.gdc.cancer.gov/, >https://portal.gdc.cancer.gov/), the Gene Expression Omnibus (GEO) (https://www.ncbi.nlm.nih.gov/geo/), and the HCCDB (http://lifeome.net/database/hccdb.html). Two HCC cohorts from the GEO database were GSE14520 and GSE76427. The screening conditions for samples in each liver cancer dataset were the same; that is, the survival time was clearly recorded, and the number of days was more than 0 days. Based on the TCGA dataset, we screened a total of 365 HCC tissues and 50 paracancerous normal tissues for further study. The number of samples meeting the screening criteria in the GSE14520 and GSE76427 datasets was 242 and 115, respectively. The dataset whose ID was HCCDB18 was obtained in HCCDB, and included 21 HCC samples that met the filtering criteria.

### Single-cell RNA sequencing data processing

The single-cell RNA sequencing (scRNA-seq) data in the GSE149614 dataset was processed using Seurat v3 (Stuart et al., 2019). The steps included quality control, normalization, scaling, dimension reduction, clustering, and visualization. Quality control standards included the number of genes in each cell <6000, the number of unique molecular identifiers in cells >100, and the distribution ratio of mitochondrial gene content in cells <15%. The log-normalization function was specified as the normalization function for all data, and the expression value of each gene in all cells was also converted to a z-score. The FindVariableFeatures function (selection.method = “vst”) in Seurat then selected the 2,000 genes with the highest standardized variance as “highly variable.” Anchors for reference assembly were calculated using the FindIntegrationAnchors function, and data were integrated using the IntegrateData function. Principal component analysis (PCA) was implemented on highly variable genes. FindNeighbors and FindClusters functions were produced to implement the shared nearest neighbor (SNN) modularity optimization-based clustering algorithm. Two dimensions were projected with Uniform Manifold Approximation and Projection (UMAP).

### Cell type annotation

The cell clusters were annotated manually according to the cluster gene marker information provided in the CellMarker database and related literature. The differentially expressed genes (DEGs) across cell types were identified by setting logfc = 0.5 and minpct = 0.35 in the FindAllMarker function in Seurat.

### Analysis of the performance of the glutamine metabolic pathway in the GSE149614 dataset

The “glutamine family amino acid metabolism (GFAAM)” process gene set was downloaded from the Kyoto Encyclopedia of Genes and Genomes (KEGG), which comprises genes involved in chemical reactions and pathways of amino acids of the glutamine family. Using the single sample gene set enrichment analysis (ssGSEA) method of the “GSVA” package, GFAAM pathway scores of different cell types in each sample of the GSE149614 dataset were calculated and visualized as a bubble diagram.

### Consensus clustering analysis

ConsensusClusterPlus was used to cluster the samples in the TCGA-LIHC cohort. The input was a matrix of genes in the GFAAM pathways expressed in the TCGA-LIHC cohort. The clustering algorithm was selected as “pam,” the distance was “pearson,” the maximum evaluated k was 10, the number of iterations was 500, and the proportion of sampling in each iteration was 80%. The optimal clustering number was judged by the cumulative distribution function (CDF) curve and verified by PCA.

### Identification, expression analysis, and correlation with biological signaling pathways of mutated GFAAM genes in molecular clusters of HCC

The data on single-nucleotide variation (SNV) and copy number variation (CNV) were extracted from TCGA. The GFAAM gene mutation in each molecular cluster was analyzed by the “maftools” package, and the expression of the mutated GFAAM gene was analyzed by the Kruskal–Wallis test. The gene set of the KEGG pathway was searched from GSEA (Subramanian et al., 2005), and ssGSEA was implemented with the “GSVA” package. The correlation between the enrichment score of the KEGG pathway and the expression of GFAAM mutant genes in each sample was analyzed by the Spearman correlation analysis.

### Analysis of the matrix content and immune infiltration in the TME

The matrix and immune contents in the TME were quantified using the “ESTIMATE” package by calculating the ssGSEA score of the two gene signatures (i.e., stromal score and immune score). At present, the algorithms developed for immune infiltration estimation are divided into two categories: gene signature-based algorithms and deconvolution-based algorithms (Li et al., 2020). CIBERSORT (Newman et al., 2015) is an algorithm based on deconvolution, which calculates the relative content of 22 infiltrating immune cells in each molecule cluster by giving a leukocyte gene signature matrix. The other deconvolution method, TIMER, quantified the abundance of six tumor-infiltrating immune cells in HCC. Two “single-sample” algorithms, microenvironment cell population (MCP) counter (Becht et al., 2016) and ssGSEA, also quantified the infiltration levels of a variety of matrix and immune cells.

### DEG identification in clusters and the construction of a risk model based on machine learning algorithms

DEGs between subtypes were screened by the criteria of log2 (fold change) | > 1 and FDR <0.05 in the “limma” package. The HCC prognostic-related genes in DEGs were identified using the “survival” package in R with *p* < 0.0001 as the threshold. Six machine learning algorithms, namely, gradient boosting machine (GBM), support vector machine (SVM), random forest, eXtreme gradient boosting (XGBoost), decision tree, and least absolute shrinkage and selection operator (LASSO), were used to develop the prognosis model. GBM is a boosting-based learning algorithm where each basic learner pays attention to the residual of the previous learner and repeats the process until the error is less than the predetermined threshold (Friedman, 2000; Shojaie et al., 2022). SVM is a two-classification model. It is unique as it runs in feature space with increasing dimensions to search the hyperplane of linearly separated positive and negative training data (Rodriguez-Perez and Bajorath, 2022). Random forest is a regression tree technique that uses bootstrap aggregation and randomization of prediction factors to achieve a high degree of prediction accuracy (Rigatti, 2017). XGBoost is an optimized GBM that has the remarkable characteristics of efficiently and flexibly dealing with missing data and assembling weak prediction models to build accurate prediction models (Chen and Guestrin, 2016). A decision tree can model nonlinear effects in the algorithmic relationship of combinatorial risk factors to produce a quantitative percentage of sensitivity to mortality (Deist et al., 2018). LASSO is a regression statistical method that enjoys some of the favorable characteristics of both subset selection and ridge regression and has been frequently used in the construction of prognostic risk models (Tibshirani, 2011). The genes involved in all six machine learning models were screened by Venn diagrams, and the constituent factors of the most concise risk model were found by stepwise regression. Multivariate Cox regression analysis gave the risk coefficient of each component gene in the risk model, and after multiplying with the expression, the risk score of each sample was obtained.

### Prediction of immunotherapy response by Tumor Immune Dysfunction and Exclusion

The Tumor Immune Dysfunction and Exclusion (TIDE) tool provides a TIDE signature, trained from treatment-naive tumor data that can predict immune checkpoint blockade (ICB) clinical response based on pre-treatment tumor profiles (Jiang et al., 2018). Higher tumor TIDE predictive scores were associated with worse ICB responses. We calculated the TIDE score for each sample in the TCGA-LIHC cohort in the TIDE web application and tested for differences among the molecular clusters and between the risk groups, respectively.

### Tissue microarray cohort

HCC tissue microarray (TMA) was performed as described previously (Liu et al., 2023).

### Cell lines and cell culture

Six HCC cell lines, e.g., MHCC97, HepG2, Hep3B, SMMC7721, HCCLM3, and Huh7, in addition to the normal-type hepatocyte L02 cell line, were purchased from the Shanghai Cell Bank of the Chinese Academy of Sciences (Shanghai, China) and cultured under routine conditions.

### Statistical analysis

All statistical analyses and tests were implemented in the R program. The Kruskal–Wallis test was performed to compare the subtype-related variables. A Student’s *t*-test and a Wilcoxon rank-sum test were used to compare variable differences between the two risk groups. Kaplan–Meier survival analyses were tested by log-rank. The accuracy of the risk model was judged by the receiver operating characteristic (ROC) curve drawn by the “timeROC” package. All statistical tests were two-sided, and statistical significance was set at 0.05.

## Results

### Cell types and GFAAM pathways distinctive of HCC

**FIGURE 1:**
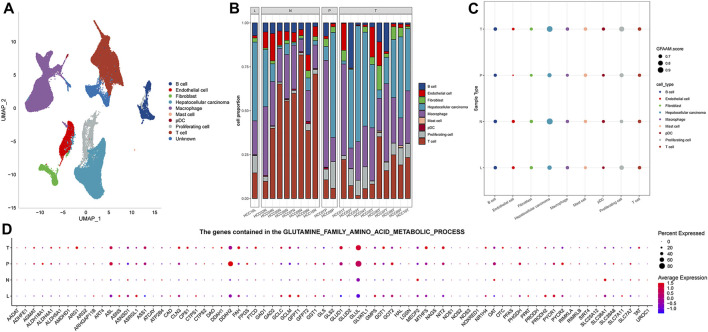
Cell types and GFAAM pathway characteristics of HCC. **(A)** Cell types are highlighted in a two-dimensional UMAP plot. **(B)** The proportion of each cell type in each sample of the GSE149614 dataset. **(C)** The enrichment score of the GFAAM pathway in nine cell types of four tissues; the size of the dots corresponded to the GFAAM score; and the color of the dots corresponded to each cell type. **(D)** Expression of GFAAM process genes in four tissue types.

The results of preprocessing and quality control of scRNA-seq data are presented in Supplementary Figure S1. High-quality cells were obtained for each sample, totaling 67,904 (Supplementary Figure S1A). By integrating all high-quality cells, we detected batch effects between the samples (Supplementary Figure S1B). To ensure the maximum extent of data recombination in different samples, batch effect correction was performed (Supplementary Figure S1C). For PCA dimensionality reduction, ElbowPlot was adopted to select all PC axes before the point with a smooth slope, where each axis was roughly distinguishable at PC 40 (Supplementary Figure S1D). Cell clustering revealed 11 cell clusters, and UMAP visualized the distribution of these clusters (Supplementary Figure S1E). Each cluster was assigned a cell-type identity according to cell-specific markers. We concluded that cluster 0 was a T cell, where CD3D, CD3E, and CD3G were highly expressed; GPC3, CD24, and MDK were highly expressed in cluster 1, which was an HCC cell; and cluster 2 was a macrophage, where CD163 and CD68 were highly expressed. There was a highly specific expression of PECAM1 in cluster 4, which was an endothelial cell. Cluster 5 was a B cell with a highly specific expression of CD19 and CD79A. MKI67 was highly expressed in cluster 6, which was a proliferating cell. Cluster 7 comprised a fibroblast, which showed a highly specific expression of ACTA2, PDGFRB, and NOTCH3. Cluster 9 had a high expression of FCER1A and LILRA4, which was a pDC. Cluster 10 consisted of a mast cell with a highly specific expression of TPSAB1 and CPA3. As the highly specific expression of typical marker genes was not detected in clusters 3 and 8, the cell types of these two clusters could not be determined (Supplementary Figure S1F). The distribution of each cell type is represented in the UMAP diagram (Figure 1A). The proportion of each cell type in each sample was evaluated. HCC and macrophages accounted for the highest proportion of MLN organizations. T cells accounted for the highest proportion in NTL organizations. Among the two PVTT organizations, the highest proportion was accounted for by HCC in one and macrophages in the other. HCC, macrophages, and T cells constituted the main cell types in the 10 PT tissues (Figure 1B). The enrichment score of the GFAAM pathway was the highest in HCC and proliferating cells of four types of tissues; however, there was no significant difference among the other seven types of cells (Figure 1C). The expression patterns and levels of GFAAM process genes varied across the four tissue types, indicating the tissue-specific heterogeneity of GFAAM molecules (Figure 1D).

### Three molecular subtypes of HCC were identified according to the expression of GFAAM process genes

**FIGURE 2:**
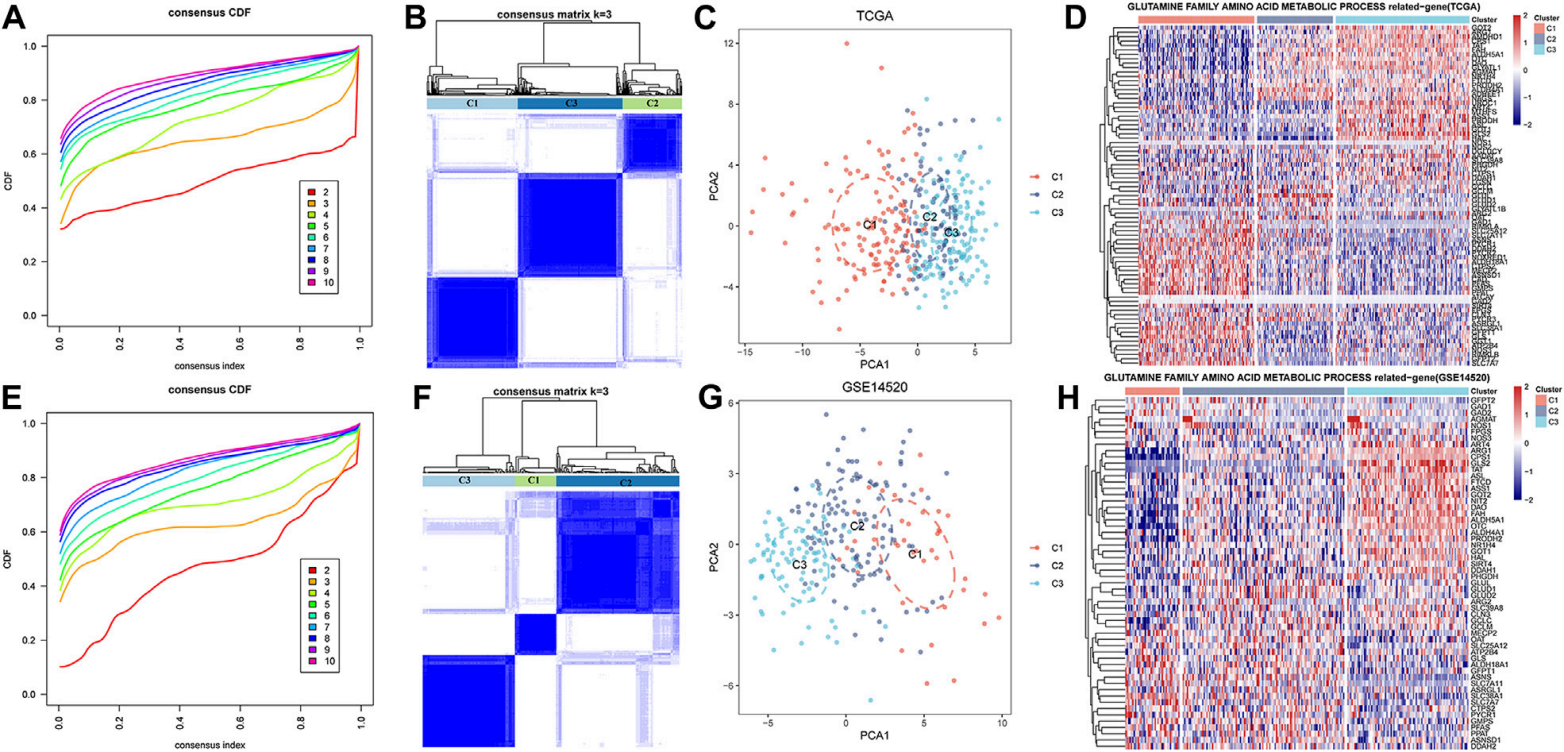
Three molecular subtypes for HCC were identified according to the expression of GFAAM process genes. **(A)** CDF curves for different subtype numbers in the TCGA-LIHC cohort. **(B)** Sample clustering for the TCGA-LIHC cohort under *k* = 3. **(C)** Three molecular subtypes in the TCGA-LIHC cohort are shown on a two-dimensional scatter plot based on PCA. **(D)** The expression of genes in the GFAAM pathway in the three molecular subtypes of the TCGA-LIHC cohort. **(E)** CDF curves for different subtype numbers in the GSE14520 database. **(F)** Sample clustering for the GSE14520 database under *k* = 3. **(G)** Three clusters in the GSE14520 database are displayed on a two-dimensional scatter plot based on PCA. **(H)** The heatmap shows the expression of genes in the GFAAM pathway in the three molecular clusters of the GSE14520 database.

Because of the heterogeneity of GFAAM molecules in HCC, we clustered the samples from the TCGA and the GSE14520 datasets according to the GFAAM process gene expression. The clustering results of the two datasets were similar, and the CDF curves of *k* = 3 showed continuity and stability; therefore, HCC was divided into three molecular subtypes (Figures 2A, B; Figures 2E, F). These three clusters were presented on a two-dimensional scatter plot based on PCA and showed different distributions (Figures 2C, G). Genes in the GFAAM pathway showed different expression patterns among the three molecular subtypes. GFAAM process genes lacking expression in C1 were overexpressed in C3, whereas those overexpressed in C1 were significantly inhibited in C3 (Figures 2D, H).

### Clinicopathological characteristics of three molecular subtypes related to the GFAAM pathway

**FIGURE 3:**
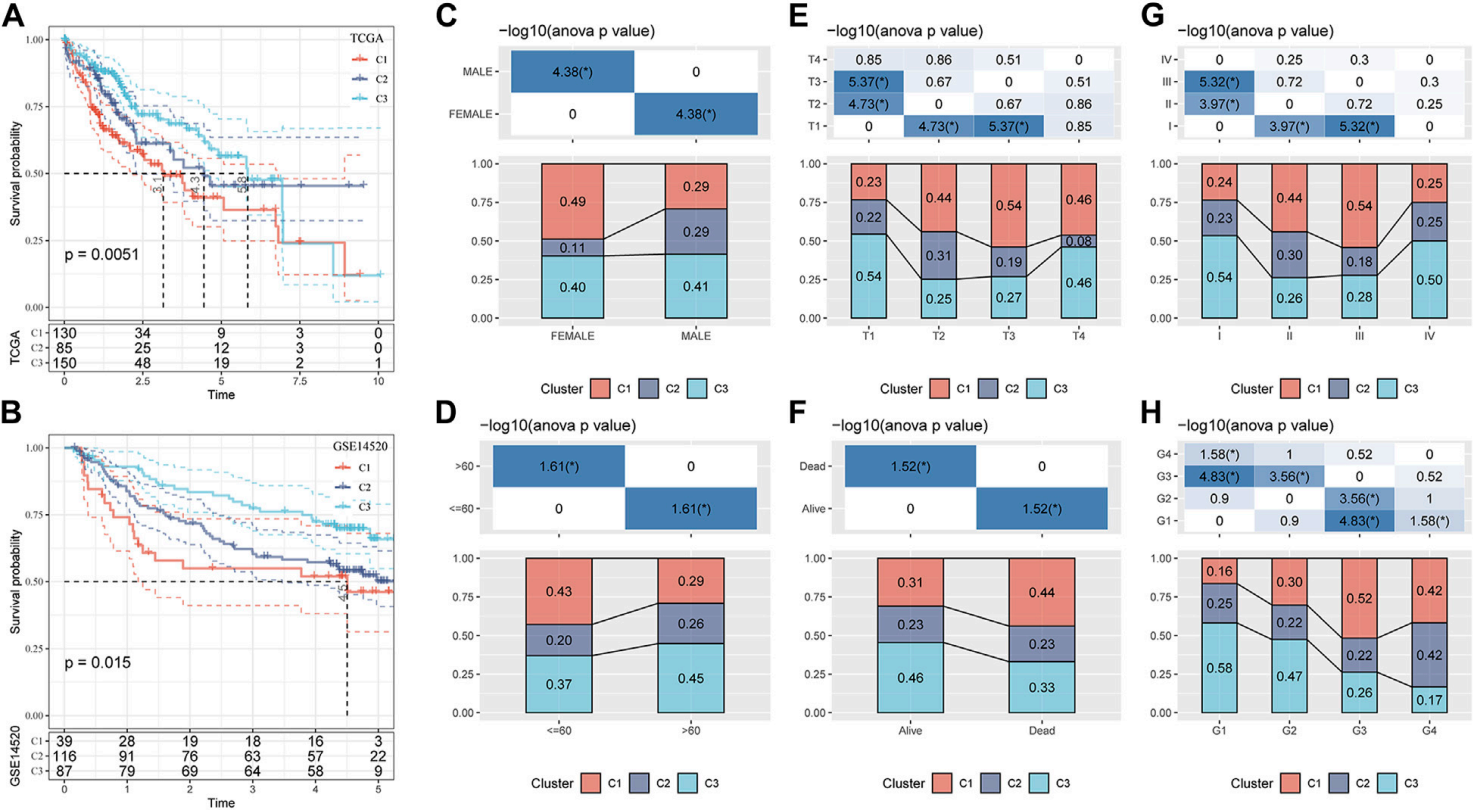
Clinicopathological characteristics of three molecular subtypes related to the GFAAM pathway. **(A)** The Kaplan–Meier survival curve of the three molecular subgroups in the TCGA-LIHC cohort. **(B)** Differences in survival rates among the three molecular subgroups in the GSE14520 dataset. **(C–H)** The proportion of the three molecular subtypes was calculated according to the clinical characteristics of sex **(C)**, age **(D)**, T stage **(E)**, survival status **(F)**, AJCC stage **(G)**, and grade **(H)**.

Based on TCGA-LIHC and the GSE14520 datasets, the differences in survival time among the three molecular subtypes were analyzed. In both datasets, the survival rates of the three molecular subgroups showed significant differences, and the trend was always C1 < C2 < C3 (Figures 3A, B). The proportion of the three molecular subtypes was calculated according to clinical characteristics, such as sex, age, T stage, survival state, AJCC stage, and grade, and it was found that the three subtypes accounted for different proportions of these clinical features, showing significant differences. There was a significant increase in the proportion of C1 and a significant decrease in the proportion of C2 in female patients compared to that in male patients. T2–T4 showed a significantly increased ratio of C1 relative to T1. The ratio of C1 in stages II and III was also significantly higher than that in stage I. C1 accounted for a significantly higher proportion in age ≤60 years, death, and G3–G4 samples than in age >60 years, survival, and G1–G2 samples (Figures 3C–H).

### Mutant GFAAM process genes and their expression and influence on signaling pathways for three molecular subtypes

**FIGURE 4:**
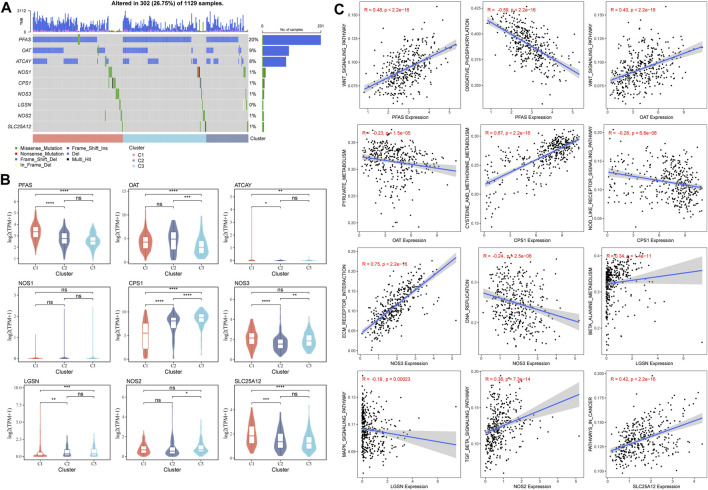
Mutant GFAAM process genes and their expression and influence on signaling pathways for three molecular subtypes. **(A)** Mutation of GFAAM process genes in the three molecular subtypes. **(B)** The mountain map shows the expression trend of nine mutated GFAAM process genes in the three molecular subgroups. **(C)** Spearman correlation analysis between the pathways shows significant enrichment differences among the three molecular subtypes and mutated GFAAM process genes.

The mutations of GFAAM process genes in the three molecular subtypes were analyzed, and nine GFAAM process gene mutations were detected. PFAS was the GFAAM process gene with the highest mutation rate, with a mutation rate of 20%; the mutation rates of OAT and ATCAY were 9% and 8%, respectively. The main mutation mode of the three genes with the highest mutation rate in the three molecular subgroups was frameshift deletion (Figure 4A). For the nine mutated GFAAM process genes, we analyzed their expression trend in the three molecular subgroups. In the violin map, we observed that the expression level of PFAS and OAT in C3 and C2 was higher than that in C1 (Figure 4B). The pathways that showed significant differences in enrichment scores among the three molecular subtypes included the Wnt signaling pathway, oxidative phosphorylation, pyruvate metabolism, cysteine, and methionine metabolism, the Nod-like receptor signaling pathway, ECM receptor interaction, DNA replication, β alanine metabolism, the MAPK signaling pathway, the TGF β signaling pathway, pathways in cancer, and other pathways. The expression of PFAS in mutated GFAAM process genes was positively correlated with the Wnt signaling pathway in these pathways and negatively correlated with oxidative phosphorylation. The expression of OAT was also positively connected with the Wnt signaling pathway and negatively connected with pyruvate metabolism. The expression of CPS1 was positively related to cysteine and methionine metabolism and negatively relevant to the Nod-like receptor signaling pathway. The pathways with the most significant positive and negative correlations with NOS3 expression were ECM receptor interaction and DNA replication, respectively. The pathways with the highest positive and negative correlations with LGSM expression were β alanine metabolism and the MAPK signaling pathway, respectively. There was a significant positive correlation between the expression of the TGF β signaling pathway and NOS2 and between cancer pathways and SLC25A12 (Figure 4C).

### Stromal and immune status of three molecular subtypes related to the GFAAM pathway

**FIGURE 5:**
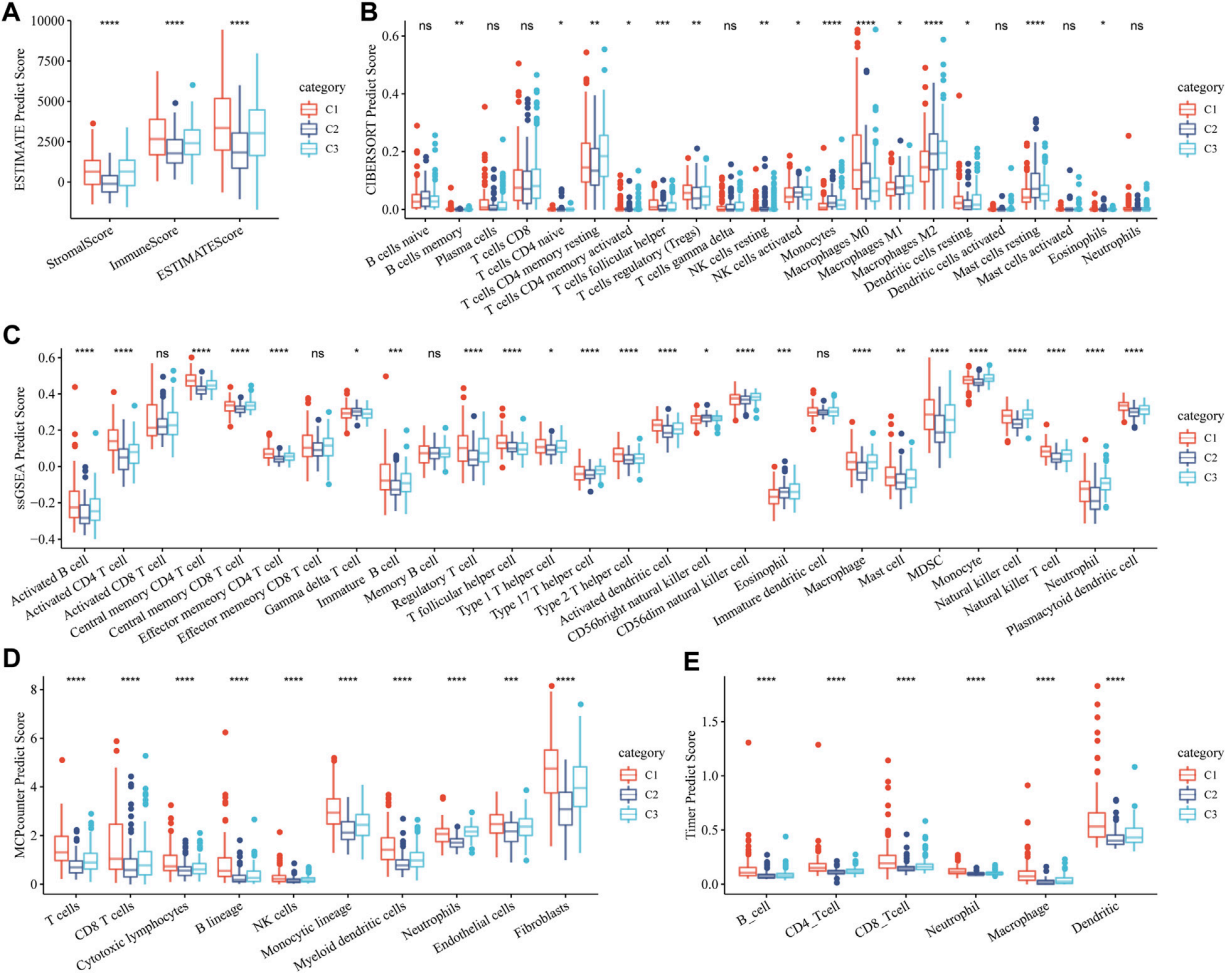
Stromal and immune status of three molecular subtypes related to the GFAAM pathway. **(A)** The differences in stromal score, immune score, and ESTIMATE score among the three subgroups. **(B)** The content of immune cells in the three molecular subgroups calculated by CIBERSORT. **(C)** Statistical differences IN the scores of 28 immune cells among the three molecular subgroups. **(D)** Differences in the abundance of stroma and immune cells concluded by MCP-counter among the three molecular subgroups. **(E)** Six immune cell scores quantified by TIMER for the three molecular subgroups. * indicates significant differences in immune scores among the subtypes; * is *p* < 0.05, ** is *p* < 0.01, *** is *p* < 0.001, and **** is *p* < 0.0001.

The stromal and immune status of the three GFAAM pathway-related molecular subtypes were characterized based on their expression profiles in the TCGA-LIHC cohort. The stromal and immune scores quantified by ESTIMATE were the lowest in C2 and significantly lower than in C1 and C3 (Figure 5A). Immune cell infiltration was calculated according to the various algorithms provided by the R program. Among the immune cells calculated by CIBERSORT, CD8^+^ T cells, resting memory CD4^+^ T cells, regulatory T cells (Tregs), activated NK cells, M0 macrophages, M1 and M2 macrophages, and resting mast cells had a high content in the three molecular subtypes. Among them, the contents of resting memory CD4^+^ T cells, M0 macrophages, M1 and M2 macrophages, and resting mast cells significantly differed among the three molecular subgroups. The contents of resting memory CD4^+^ T cells, activated NK cells, M1 macrophages, M2 macrophages, and resting mast cells in C3 were significantly higher than those in C1. The contents of Tregs and M0 macrophages in C1 were significantly higher than those in C3 (Figure 5B). The infiltration score trend of most of the 28 immune cells assessed using ssGSEA in the three molecular subtypes was consistent with the performance of their immune score assessed by ESTIMATE, among them multiple types of B-cell subsets (activated and immature B cells), T-cell subsets (activated CD4^+^ T, central memory CD4^+^ T, central memory CD8^+^ T, effector memory CD4^+^ T, regulatory T, type 1 helper, type 2 helper, type 17 helper, and natural killer T cells), activated dendritic cell, CD56 dim natural killer cell, macrophage, mast cell, myeloid-derived suppressor cell (MDSC), monocyte, natural killer cell, neutrophil, and plasmacytoid dendritic cell (Figure 5C). The stromal and immune cells evaluated by MCP-counter and TIMER also showed significant differences among the three molecular subgroups, and the infiltration abundance in C2 and C3 was significantly lower than that in C1 (Figures 5D, E).

### Construction of a risk model using six machine learning algorithms and verification in verification cohorts

**FIGURE 6:**
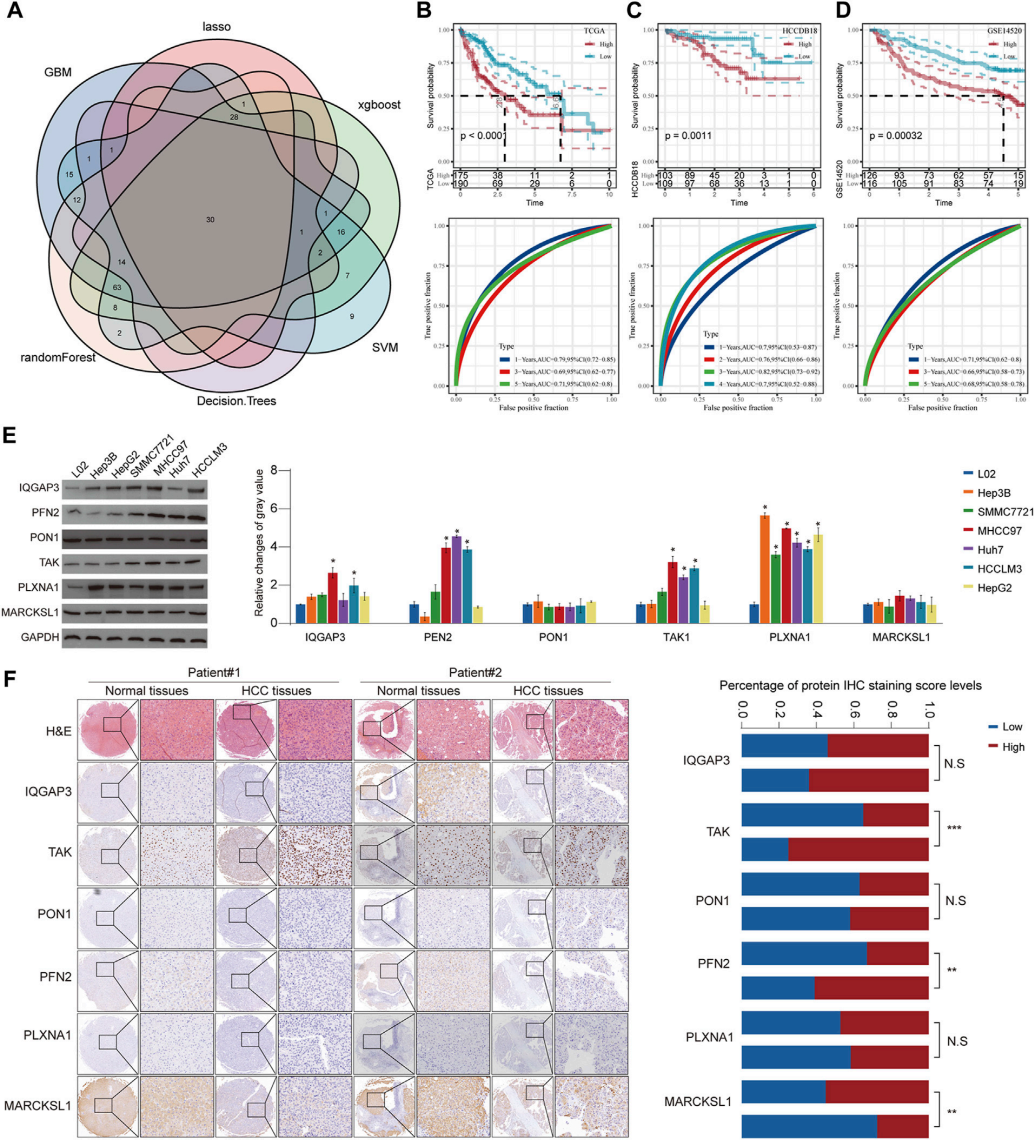
Construction of a risk model using six machine learning algorithms and verification in verification cohorts. **(A)** Venn diagram presents HCC prognosis-related genes that were involved in all six machine learning models. **(B)** Prognostic prediction and performance evaluation of the risk evaluator in the TCGA-LIHC dataset. **(C)** The relationship between the risk evaluator and sample survival and THE ROC curve in the HCCDB18 dataset. **(D)** Kaplan–Meier curve and ROC curve of risk score in the GSE14520 dataset. **(E)** The protein level of six evaluators in HCC cell lines and normal liver cells determined by Western blot. **(F)** Representative images of IHC staining of the risk evaluator in HCC TMA cohorts (left). The IHC score of six proteins in HCC tissues and adjacent normal tissues was further quantified (right). * is *p* < 0.05, ** is *p* < 0.01, *** is *p* < 0.001, and **** is *p* < 0.0001.

Although HCC has been divided into three subgroups according to GFAAM process genes, it does not quantify the state of the individual. Therefore, we constructed a risk model based on the characteristic genes of the GFAAM pathway-related subtypes, that is, DEGs, among the three subgroups. By identifying the DEGs between C1, C2, and C3 and the DEGs between C2 and C3, a total of 2,250 characteristic genes related to the GFAAM pathway were obtained (Supplementary Figure S2). Among the 2,250 genes, 211 were prognostic genes that met the screening criteria of the univariate COX regression analysis. Six machine learning algorithms were used to verify 211 genes, and 30 genes were found to be significant in all the machine learning models (Figure 6A). Using stepped-regression analysis, six genes were obtained from the 30 genes and used to construct the risk evaluator: Risk Score = −0.231×PLXNA1+0.192×MARCKSL1+0.318×IQGAP3+ 0.141×PFN2-0.102×PON1+0.157×TKT.

The risk evaluator calculated the risk score of each sample in the TCGA-LIHC (training set), HCCDB18 (validation set 1), and GSE14520 datasets (validation set 2). After standardization by z-score, the high- and low-risk groups were separated by linking the risk score to survival data to determine the prognosis of the sample. In the TCGA-LIHC dataset, high-risk samples had significant survival advantages over low-risk samples. The ROC curve showed that the risk evaluator effectively predicted the survival of patients in the TCGA-LIHC dataset, with the 1-year area under the curve (AUC) = 0.79, 3-year AUC = 0.69, and 5-year AUC = 0.71 (Figure 6B). Similarly, in verification sets 1 and 2, the Kaplan–Meier curves of high- and low-risk samples were significantly separated, and the survival results of high-risk samples were significantly better than those of low-risk samples. The AUC value of the ROC curve for the HCCDB18 cohort (determined annually) was high (>0.7) for 1–4 years. The ROC curve for the GSE14520 dataset showed effective predictive ability at 1, 3, and 5 years (Figures 6C, D). Furthermore, we evaluated the protein expression level of the abovementioned risk evaluator in several HCC cells (Figure 6E) and validated it in a relatively large sample of HCC tissue microarray (Figure 6F). The results showed that the expressions of TAK and PFN2 were markedly upregulated, whereas MARCKSL1 expression was downregulated in HCC tissues compared with that in surrounding non-tumorous tissues, indicating the promising prognostic value of the abovementioned model.

### Characteristics of potential factors affecting the prognosis of HCC

**FIGURE 7:**
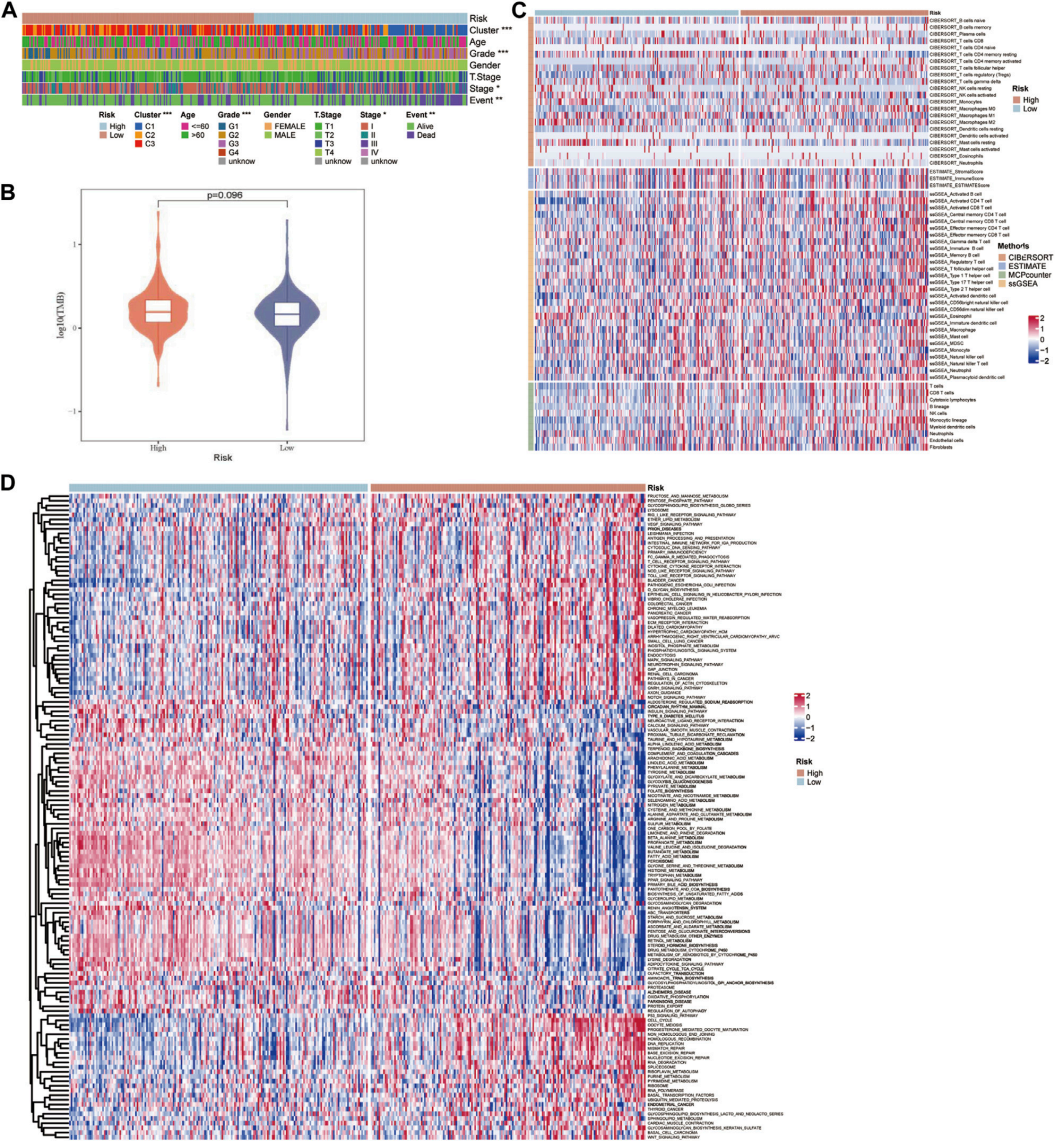
Characteristics of potential factors affecting the prognosis of HCC. **(A)** The distribution heatmap of clinicopathological features in high-risk and low-risk groups of the TCGA-LIHC dataset. **(B)** The difference in tumor mutation burden between the high-risk and low-risk groups. **(C)** The heatmap summarizes the immune microenvironment indexes in the high-risk and low-risk groups. **(D)** The signaling pathway shows significant enrichment differences between the high-risk and low-risk groups.

The TCGA-LIHC data were employed to explore the differences in potential factors affecting the prognosis of HCC between the high- and low-risk groups, which included clinicopathological features, somatic mutations, TME index, and signaling pathways. There were significant differences in the distribution of molecular subtypes related to the GFAAM pathway and the proportion of grade, AJCC stage, and survival state between the high- and low-risk groups. In the high-risk group, the dominant GFAAM pathway-related molecular subtype was C1, the grade was G3, the AJCC stage was stage III, and the proportion of deceased patients was also high. In the low-risk group, the dominant molecular subtypes of the GFAAM pathway, grade, and AJCC stage were C3, G2, and stage Ⅰ, respectively, and the proportion in samples from live patients was much higher than that from deceased patients (Figure 7A). As shown in Figure 7B, the high-risk group had a higher tumor mutation load relative to the low-risk group, but the difference was not significant (Figure 7B). In the heatmap representing the immune landscape, immune indicators included the immune score, indicating the level of immune cell infiltration was higher in the high-risk group (Figure 7C). In terms of enriched pathway scores, many immune-regulatory and carcinogenic signaling pathways were significantly upregulated in the high-risk group compared with those in the low-risk group, whereas a considerable number of metabolic pathways were significantly inhibited in the high-risk group compared with those in the low-risk group (Figure 7D).

### Indicators related to the immunotherapy response of GFAAM pathway-related molecular subtypes and risk groups

**FIGURE 8:**
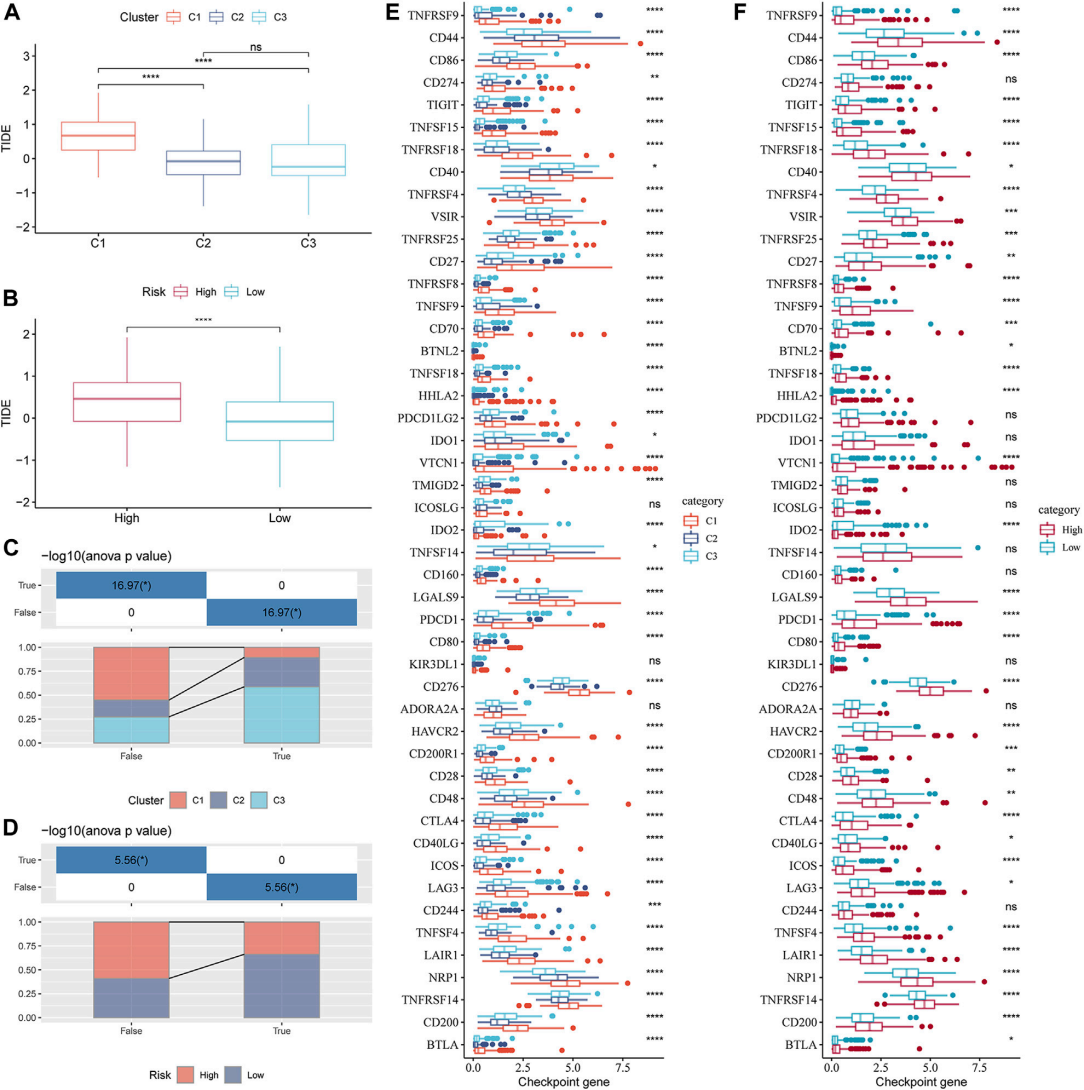
Indicators related to the immunotherapy response of GFAAM pathway-related molecular subtypes and risk groups. **(A)** TIDE score differences among molecular subtypes related to the GFAAM pathway in the TCGA-LIHC cohort. **(B)** The comparison of TIDE scores between the high-risk group and the low-risk group defined by the risk evaluator in the TCGA-LIHC cohort. **(C)** The proportion of each GFAAM pathway-related molecular subtype in the ICB treatment response and non-response groups. **(D)** The proportion of high-risk and low-risk samples in the ICB treatment response and non-response groups. **(E)** Differences in the expression of immune checkpoint molecules among molecular subtypes related to the GFAAM pathway. **(F)** Comparison of immune checkpoint molecular expression between the high-risk and low-risk groups. Statistically significant differences are marked by *, * is *p* < 0.05, ** is *p* < 0.01, *** is *p* < 0.001, and **** is *p* < 0.0001.

Understanding the potential indicators that affect ICB treatment response can help select patients who can benefit from it. For the samples in the TCGA-LIHC cohort, the TIDE scores of C2 and C3 were significantly lower than those of C1, and the response rates of C2 and C3 to ICB therapy were lower than those of C1 (Figures 8A, C). The TIDE score of the low-risk group, as defined by the risk evaluator, was significantly lower than that of the high-risk group, and the response rate of the low-risk group to ICB treatment was higher than that of the high-risk group (Figures 8B, D). The TIDE score, response rate of ICB treatment, and expression of immune checkpoint molecules (another important indicator of ICB treatment response) were compared between molecular subtypes and risk groups. Numerous immune checkpoint molecules showed significantly higher expression levels in C1 than in C2 or C3 (Figure 8E). The levels of most immune checkpoint molecules in the high-risk group were significantly higher than those in the low-risk group (Figure 8F). In terms of the abovementioned indicators related to ICB therapy, C1 was the least suitable for ICB therapy among the three GFAAM pathway-related molecular subgroups. Moreover, the potential effect of ICB therapy in the high-risk group was not as beneficial as that in the low-risk group.

## Discussion

The liver is a metabolic center with a unique immunosuppressive microenvironment (Jenne and Kubes, 2013; Scagliola et al., 2023). Metabolic disorders are considered an important driving force in the pathogenesis of HCC, leading to profound changes in the TME (Leone and Powell, 2020; Chen et al., 2023). Glutamine is the primary substrate that supports bioenergetics and biosynthesis activity in cancer cells and provides them with supplementary energy (Delgir et al., 2021). Increased glutamine catabolism is one of the critical metabolic features of cancer cells (Du et al., 2022), whose fate varies with a range of parameters, such as their tissue of origin, the genetic aberration that drives them, and the TME (Cluntun et al., 2017). There are few studies simultaneously focusing on glutamine metabolism, TME, cancer tissue subtypes, and related genetic mutation disturbances, which is a more meaningful research direction. In this study, we first identified 10 cell types in HCC. The overall level of the GFAAM pathway did not show significant differences among these types of cells but showed significant heterogeneity among different HCC tissue types. Therefore, we classified HCC molecular subtypes according to the genes in the GFAAM pathway and simultaneously studied the clinicopathological characteristics, GFAAM pathway gene mutations, and TME of each subtype. We also used the newly obtained molecular typing to develop marker combinations (based on machine learning) to evaluate the prognosis, mutation pattern, TME, and response to the ICB treatment of HCC.

In the molecular classification of HCC, we identified three molecular subtypes related to the GFAAM pathway: C1, C2, and C3. These subtypes have great heterogeneity, showing different clinicopathological features, GFAAM process gene mutations, and TME features. C1 had the worst prognosis; patients aged 60 years and below; middle-, late-, and high-grade samples; and deceased patients. C1 also showed higher immune scores for B, T, CD4^+^ T, and CD8 + T cells, macrophages, MDSC, Treg cells, dendritic cells, and fibroblasts than C2 and C3. Cytotoxic CD8^+^ T, CD4^+^ T, and NK cells work together to maintain immune surveillance, whereas the abundant immune cells in HCC, such as MDSC, Tregs, and tumor-associated macrophages, help the immune evasion to accelerate tumor progression (Chen et al., 2023). Ma et al. (2022) showed that reprogramming of glutamine metabolism plays a key role in the survival of immune cells in the TME, largely due to the fact that there is competition for glutamine uptake between these cells in the TME (31). It has been shown that in clear cell renal cell carcinoma, competitive depletion of glutamine by tumor cells leads to local deprivation of extracellular glutamine, which in turn activates the production of HIF-1α and induces cytokine secretion from tumor-infiltrating macrophages. In addition, in triple-negative breast cancer, tumor cells have been found to competitively uptake glutamine from the TME, which in turn affects glutamine availability to tumor-infiltrating T lymphocytes and influences their antitumor immune response (Fu et al., 2019). These results suggest that in the TME of HCC, abnormal metabolism of glutamine directly affects the degree of infiltration of tumor immune cells, which in turn affects the antitumor activity of immune cells.

Our results agree with the development trend of cancer because the tumor-antagonistic immune cells within the TME tend to target and kill cancer cells in the early stage of tumorigenesis; however, as cancer continues to develop, cancer cells eventually escape immune surveillance. In the immune escape phase, tumor cells continue to grow and proliferate uncontrolled and are no longer restricted by host immunity (Gajewski et al., 2013; Vinay et al., 2015). In this study, there were more mid-late and high-grade samples in C1, indicating a higher malignant degree where immune escape occurred. This was further confirmed by the results of the TIDE analysis. C1 showed a significantly higher TIDE score compared to C2 and C3, which indicated a higher immune escape potential.

Models of specific cancer diagnoses and prognostic indicators constructed from subtype studies have been widely used in clinical research, usually relying on machine learning algorithms (Zhao et al., 2019). In this study, we used six machine learning algorithms to construct a model of prognosis-related DEGs between subtypes, identified 30 genes involved in each machine learning model, and captured six of them to develop a risk evaluator. Each gene in the risk evaluator, PLXNA1 (Ho, 1988), MARCKSL1 (Egeland et al., 2019), IQGAP3 (Leone et al., 2021), PFN2 (Cui et al., 2016), PON1 (Bobin-Dubigeon et al., 2012), and TAK (Li et al., 2022), has been reported to be associated with the prognosis or progression of cancer. Here, the risk evaluator developed using these six genes was associated with potential factors affecting the prognosis of HCC, such as clinicopathological features, somatic mutations, tumor microenvironment indicators, and signaling pathways. These features had significant predictive value for the prognosis of HCC and the response to ICB treatment. However, some limitations should also be noted. The data for our study were extracted only from the TCGA, GEO, and HCCDB18 databases, which are only at the level of bioinformatics. Further *in vivo* and *in vitro* validation tests are needed to verify that our prognostic model is excellent for prediction. Furthermore, the molecular mechanisms involved in glutamine metabolism in HCC still require more in-depth studies.

In conclusion, we identified 10 cell types and three GFAAM pathway-related molecular subtypes with clinical and molecular heterogeneity in HCC and constructed a model for the prognostic prediction of immunotherapeutic response among the subtypes. Our study offers new perspectives on the use of glutamine metabolism in clinical research on HCC.

## Data Availability

The original contributions presented in the study are included in the article/Supplementary Material; further inquiries can be directed to the corresponding author.
