# Supplementary figures and images for: Identification of a prognostic evaluator from glutamine metabolic heterogeneity studies within and between tissues in hepatocellular carcinoma
Source: Front Pharmacol. 2023 Oct 26;14:1241677. doi: 10.3389/fphar.2023.1241677 (PMC10637396; doi:10.3389/fphar.2023.1241677)

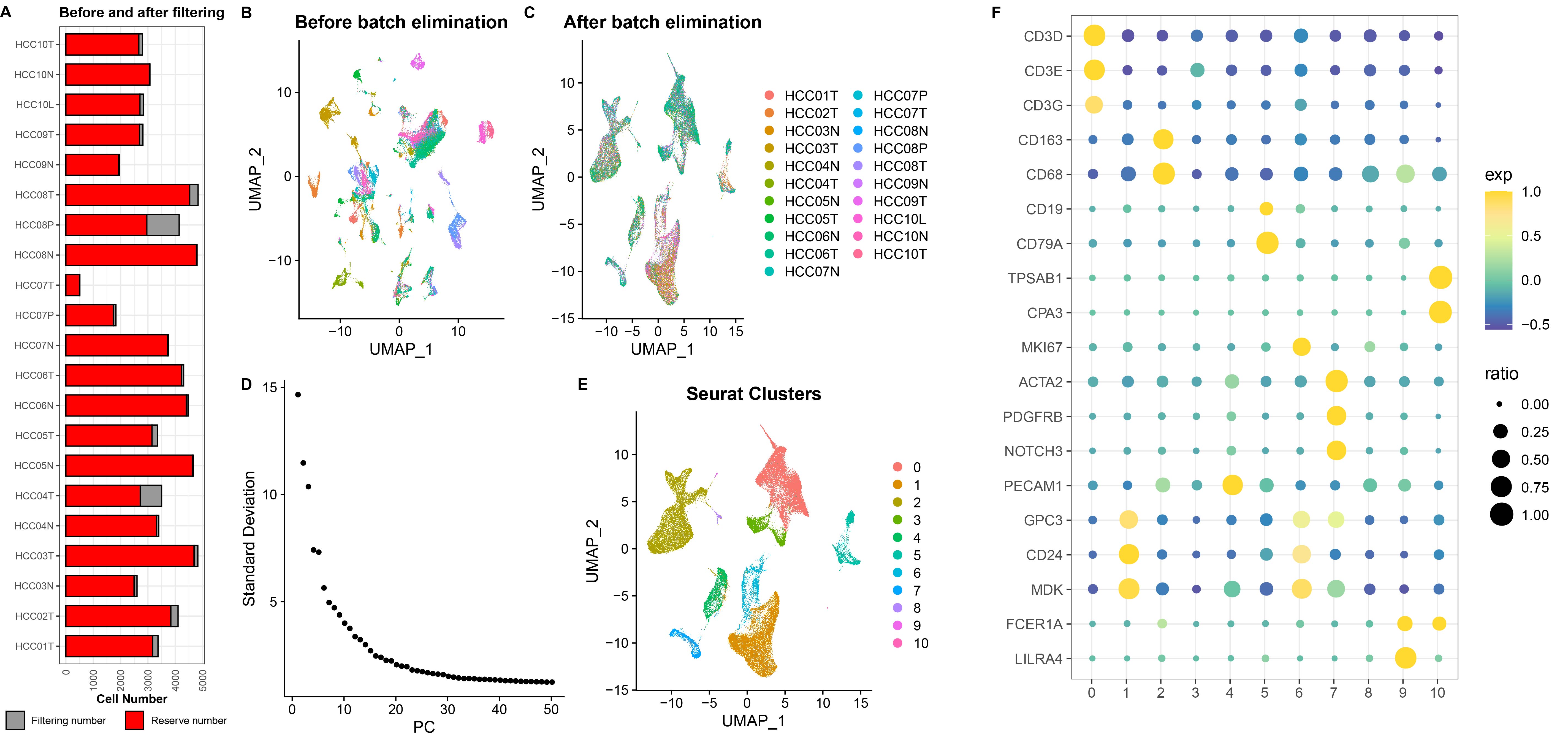

Supplement: Supplementary file 1 [file Image1.JPEG]

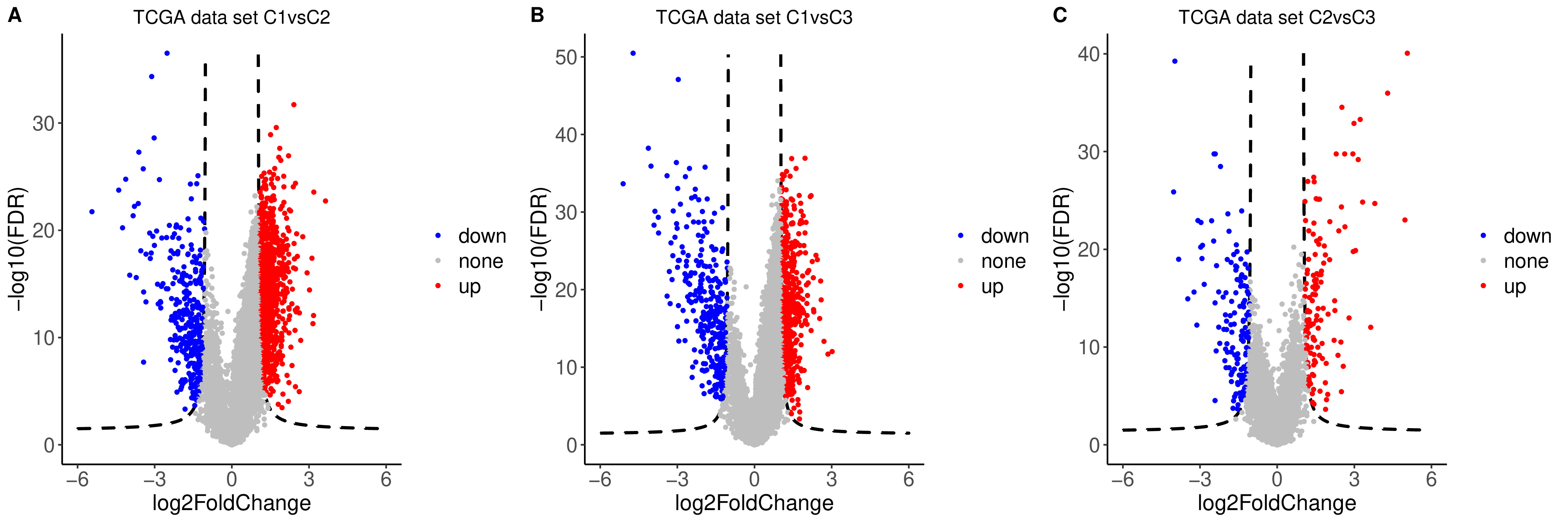

Supplement: Supplementary file 2 [file Image2.JPEG]
