# Supplementary material for: Identification of a prognostic evaluator from glutamine metabolic heterogeneity studies within and between tissues in hepatocellular carcinoma
Source: Front Pharmacol. 2023 Oct 26;14:1241677. doi: 10.3389/fphar.2023.1241677 (PMC10637396; doi:10.3389/fphar.2023.1241677)
Supplement: Supplementary file 3 [file DataSheet1.docx]

**Description of Supplementary Figures**

**Figure S1 Quality control and cell clustering of scRNA-seq data in GSE149614**

(A) Screening of high-quality cells for each sample in GSE149614 dataset. (B) The distribution of samples after the integration of all high-quality cells. (C) The sample distribution after correcting the batch effect. (D) The selection of appropriate PC for clustering. (E) UMAP visualized 11 cell clusters generated by clustering. (F) The expression of marker genes that assign cell type identity to each cluster.

**Figure S2 Identification of characteristic genes for GFAAM pathway-associated subtypes**

(A) DEGs between C1 and C2. (B) DEGs between C1 and C3. (C) DEGs between C2 and C3.
